# Surviving Capnocytophaga Canimorsus Septic Shock: Intertwining a Challenging Diagnosis with Prompt Treatment

**DOI:** 10.3390/diagnostics12020260

**Published:** 2022-01-20

**Authors:** Fulvio Nisi, Andrea Dipasquale, Elena Costantini, Enrico Giustiniano, Umberto Ripani, Maurizio Cecconi

**Affiliations:** 1 Department of Anesthesia and Intensive Care Units, IRCCS Humanitas Research Hospital, 20089 Milan, Italy; elena.costantini@humanitas.it (E.C.); enrico.giustiniano@humanitas.it (E.G.); maurizio.cecconi@hunimed.eu (M.C.); 2 Department of Biomedical Sciences, Humanitas University, Pieve Emanuele, 20090 Milan, Italy; andrea.dipasquale@humanitas.it; 3 Department of Internal Medicine, Humanitas Clinical and Research Center—IRCCS, Rozzano, 20089 Milan, Italy; 4 Division of Clinic Anaesthesia, Department of Emergency Hospital Riuniti, Conca Street 71, 60126 Ancona, Italy; umberto.ripani@ospedaliriuniti.marche.it

**Keywords:** critical care medicine, sepsis, interesting cases in emergency medicine

## Abstract

*Capnocytophaga canimorsus* is zoonotic agent isolated from humans bitten by dogs or cats. Although rare, severe infection usually affects male patients over the age of 50, asplenic or immunocompromised. Diagnosis is often challenging, often missing a history of contact with dogs or pre-existing wounds. Mortality rate is extremely high, since infection can lead to fulminant sepsis. We report a case of a patient admitted to ED for septic shock of unknown origin. Severe sepsis developed since our patient was asplenic and possessed multiple comorbidities. Due to hypoxia and respiratory failure, the patient was promptly intubated and mechanically ventilated. Supportive treatment for hemodynamic shock was administered. Cultures were obtained in the ED and empiric antibiotic therapy with piperacillin/tazobactam was started, aiming at infection control. As for source identification, common infectious etiologies, SARS-CoV-2 swab, bronchoalveolar lavage and urine cultures were negative. Blood cultures proved Gram-negative rods after 12 h incubation and *C. canimorsus* was identified on day 4. During ICU stay, clinical conditions gradually improved, and source control proved to be effective. Culture samples collection and starting empiric antibiotic treatment are the essential points in ensuring patient survival, especially in sepsis or septic shock of unknown origin or uncommon etiology, as in our case. Why should an emergency physician be aware of this? *C. canimorsus* bacteremia is rare and difficult to diagnose. Although considering patient history in such cases is crucial, laboratory results are often delayed. Hence, the chance of survival is dependent on prompt culture samples collection and start of empiric antibiotic treatment, along with supportive treatment.

## 1. Introduction

We report the case of a patient admitted to the emergency department (ED) with septic shock from an uncommon agent, *Capnocytophaga canimorsus. C. canimorsus* is a zoonotic agent isolated from humans bitten by dog or cat. It rarely leads to infection in humans, with an infection rate between 0.5 and 0.67 cases per million [1]. Infection usually affects men over the age of 50, and it is more common and severe in asplenic or immunocompromised patients. Diagnosis is often challenging, often missing a history of contact with dogs or pre-existing wounds. Mortality rate is extremely high [2], since infection can lead to fulminant sepsis. Hence, survival is strictly related to prompt empiric treatment.

## 2. Case Report

A 63-year-old man presented to the emergency department (ED) with fever (T 38 °C), cough, and asthenia for a few days. His medical history was notable for coronary heart disease and atrial fibrillation, which required an implantable cardioverter defibrillator, chronic obstructive pulmonary disease, a smoking habit, previous splenectomy for immune thrombocytopenia (ITP), and chronic myelomonocytic leukemia in treatment with hydroxycarbamide.

In the ED his vital signs quickly deteriorated, revealing a drowsiness, tachycardia with 140 bpm, blood pressure of 50/30 mmHg, temperature of 37 °C, and hypoxia (pO2 of 50 mmHg to blood gas analysis). Blood samples showed C-reactive protein 14.57 mg/dL, white blood count (WBC) 28.440 10^3^/mm^3^ (93% neutrophils), procalcitonin (PCT) 151 ng/mL, creatinine 2.72 mg/dL, and total bilirubin 1.9 mg/dL.

**Figure 1:**
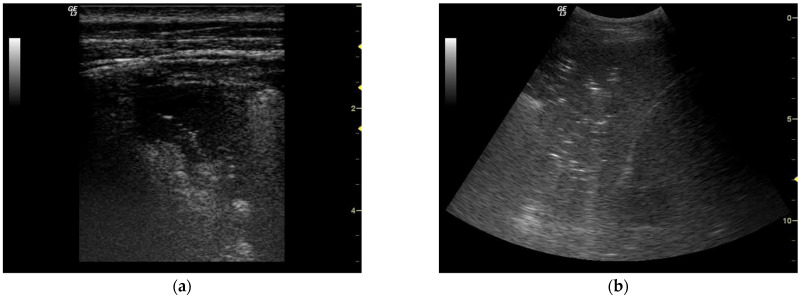
Lung ultrasounds: (**a**) Linear probe. Left superior lobe anterior segment of the lung. Subpleural lobar consolidation with irregular margins; the area of consolidation includes sonographic air bronchogram. (**b**) Curvilinear probe. Right posterior lower lobe posterior basal segment of the lung. Image of tissue echogenicity associated with aerial bronchogram (represented by hyperechoic punctuate images) strongly suggestive for alveolar syndrome.

A total-body ultrasound (US) assessment was performed. Lung US revealed bilateral basal consolidations (Figure 1) and moderate pleural effusions. Abdominal US was negative for effusions of free fluids, whilst scans performed on the left hypochondrium raised doubts concerning reliability since the spleen was not clearly visible. Thus, a CT scan was required.

A non-contrast chest CT scan confirmed bilateral basal consolidations, CT scans of brain, abdomen, and pelvis were negative for infective findings, and an abdominal CT scan confirmed splenectomy. Due to hypoxia and respiratory failure, 3 h after his arrival in the ED the patient was intubated and mechanically ventilated; then, he was transferred to intensive care for the treatment of septic shock.

Persistent hypotension required fluids and hemodynamic support with noradrenaline (peak dosage 0.8 mcg kg^−1^ min^−1^). Cultures were obtained in the ED and empiric antibiotic therapy with piperacillin/tazobactam was started, aiming at infection control. As for source identification, common infectious etiologies (*Cytomegalovirus*, *Mycoplasma pneumoniae*, *Epstein–Barr virus*, *Chlamydia pneumoniae*, and *Legionella pneumophila*) were excluded. A SARS-CoV-2 test was repeated and confirmed to be negative. Bronchoalveolar lavage and urine cultures were negative. Meanwhile, after 11 h of incubation, two aerobic blood cultures isolated a Gram-negative rod. After 24–36 h, small colonies grew in a chocolate agar under an enriched 5% CO2 environment. Using a matrix-assisted laser desorption/ionization time-of-flight mass spectrometry (MALDI-TOF) Vitek MS^®^ (bioMérieux, Vila Nova de Gaia, Portugal), *C. canimorsus* was identified on blood culture on day 4. Further history analysis revealed that the patient lived with his two dogs, although no wound or scar was found after re-examination of the skin.

During the patient’s ICU stay, clinical conditions gradually improved, and source control proved to be effective since *C. canimorsus* was sensitive to the empiric antibiotic therapy. Lung US confirmed the resolution of respiratory impairment. On day 7 the patient was weaned from respiratory support, and discharge from the internal medicine ward was possible on day 15. Blood cultures repeated 20 days after admission were negative, and the patient was discharged alive from the hospital. After discharge from the ICU, written informed consent and ethical approval were obtained for clinical case publication.

## 3. Discussion

*Capnocytophaga canimorsus* is a commensal bacterium in the oral flora of dogs and cats; the bacterium is a zoonotic agent and has been isolated from humans infected by dog or cat bites, scratches, licks or simply exposure to dogs or cats. Classified as a facultative anaerobic, it is a Gram-negative rod (1–4 mm long), fusiform or filamentous, gliding bacteria closely related to Fusobacterium and Bacteroides species; it grows slowly on blood, incubated for at least 5 days, up to 14 days [2].

*C. canimorsus* is able to avoid the immune system in the early stages of infection by means of the downregulation of TLR4 and the proinflammatory signaling cascade; it is also resistant against phagocytosis and killing. Considered to be less virulent in healthy people, *C. canimorsus* could be seriously harmful in immunocompromised subjects such as patients with asplenia, a long history of alcohol abuse, cirrhosis, immunosuppressive therapy, hemochromatosis, beta-talassemia major and a cigarette-smoking habit. Our patient presented some of these risk factors. Nevertheless, cases among immunocompetent persons have been described [3].

Initially, patients may show local lesions related to animal bites, without significant signs of inflammation, or localized cellulitis, pain at the site of injury, lymphangitis and regional lymphadenopathy. The initial symptoms of septicemia are fever, chills, myalgia, vomiting, diarrhea, abdominal pain, dyspnea and mental confusion. A fulminant and severe course of the infection in immunocompromised persons is characterized by sepsis, meningitis, osteomyelitis, peritonitis, endocarditis, pneumonia, disseminated intravascular coagulation (DIC), and fulminant purpura; they also been observed in healthy patients. The median time from exposure to sepsis is 3 days, and 7 days for meningitis [4].

Due to unspecific presentation and slow growth on cultures, the diagnosis and treatment of *C canimorsus* bacteremia are often challenging, unless the history of a dog bite is clear. Other studies have reported similar cases; however, in these cases, a dog bite was visible on presentation, unlike in the present case. Indeed, although specific bacteremia after an animal bite such as *C. canimorsus* or *Pasteurella multocida* could be suspected, the lack of any skin lesion as a route of infection should not be employed as a rule-out criteria. Currently, the MALDI-TOF MS method is the gold standard for detecting bacteria in blood samples. Nevertheless, Gram-stain exam under the microscope may reveal multiple Gram-negative, extracellular, fusiform rods and several intracellular copies of the pathogen in neutrophils even before MALDI-TOF, thus leading clinicians to suspect the presence of *C. canimorsus* thanks to the observation of toxic granulation in the cytoplasm of neutrophils in peripheral blood smear. Indeed, matching examination of peripheral blood smears with patient history and clinical examination might certainly assist diagnosis, and guide prompt empiric treatment in patients with septicemia following close animal contact [5].

*C. canimorsus* is generally sensitive to penicillin, third-generation cephalosporins, carbapenems, clindamycin, doxycycline, chloramphenicol, macrolides, rifampin, and fluorochinolones, whilst it is considered to be resistant to aztreonam, trimethoprim, Fosfomycin, and aminoglycozides. The length of treatment varies between reports, from 14 to 21 days. The literature presents a few cases of patients infected by *C. canimorsus* who died due to complications caused by the septic state, often due to misunderstood diagnosis or late treatment. According to the literature, mortality from *C. canimorsus* ranges from 10% to 30% [6], with the mortality rate of severe sepsis being as high as 26%. Early culture sample collection, pertinent and timely-administered antibiotic therapy, and intensive support treatment, as in this case, decrease the risk of complications and increase survival, especially in patients with coexisting risk factors or comorbidities [7].

## 4. Conclusions

*C. canimorsus* bacteremia is rare and difficult to diagnose, and lab cultures require time to provide useful findings. Although evaluating patient history in such cases is crucial, especially when considering that most patients have no signs of infection at the wound site, the key point is that the highest chance of survival is dependent on prompt culture samples collection and start of empiric antibiotic treatment, along with supportive treatment. Indeed, culture samples collection for infection identification and starting empiric antibiotic treatment for source control are among the main focuses of the Surviving Sepsis Campaign recommendations, and they are the essential points in ensuring patient survival, especially in sepsis or septic shock of unknown origin or uncommon etiology, such as in our case.
